# ASO Author Reflection: Learning Curves in Robotic Partial Nephrectomy—Not Only the Surgeon Counts

**DOI:** 10.1245/s10434-020-08866-z

**Published:** 2020-07-22

**Authors:** Philip Zeuschner, Matthias Saar, Martin Janssen

**Affiliations:** 1 Department of Urology and Pediatric Urology, Saarland University, Homburg/Saar, Germany; 2 Present Address: Department of Urology and Pediatric Urology, University Hospital of Munster, Munster, Germany

## Past

Robot-assisted partial nephrectomy (RAPN) has rapidly evolved into a standard technique in high-volume centers. To improve surgical outcomes, especially the learning curve of the robotic surgeon has extensively been analyzed so far.^1^ Furthermore, the annual caseload has been linked with better outcomes, referring to the learning curve of a department.^2^ However, data about the role of the bedside assistant is scarce and inconclusive.^3^ For this reason, we performed a comparative analysis to assess the impact of the learning curve of the department, surgeon, and bedside assistant, as well as of patient-related factors on perioperative outcomes of RAPN.^4^

## Present

Our first 500 transperitoneal RAPN were retrospectively analyzed. The experience “EXP” was defined as the current number of RAPNs conducted by either (1) the department, (2) the surgeon, or (3) the assistant. In multiple regression analysis, not only EXP of the surgeon and EXP of the department, but also EXP of the bedside assistant had a significant impact on perioperative outcomes. Higher EXP of the assistant was linked to shorter operating times, lower conversion, and higher success rates (defined as MIC: positive surgical margin, warm ischemia time, complications). Consequently, the impact of the bed-side assistant in RAPN has clearly been underestimated so far. Tumor complexity (PADUA score) impacted most perioperative outcome parameters and was thereby the most important patient-related factor. Perioperative outcomes significantly improved with EXP > 100 for the department, EXP > 35 for the surgeon, and EXP > 15 for the assistant.

## Future

Although the fundamental need for bedside assistants in robotic surgery has been questioned recently, the complexity of robotic partial nephrectomy renders *all* participants of RAPN highly important. Training strategies in robotic surgery should not only focus on the surgeon but also on the department and bed-side assistants, which is currently clearly underdeveloped for RAPN.
